# In Vitro Evaluation of *Candida* spp. and *Staphylococcus aureus* Sensitivity to 450 nm Diode Laser-Mediated Antimicrobial Photodynamic Therapy with Curcumin and Riboflavin

**DOI:** 10.3390/ijms26125645

**Published:** 2025-06-12

**Authors:** Marcin Tkaczyk, Anna Mertas, Anna Kuśka-Kiełbratowska, Jakub Fiegler-Rudol, Elżbieta Bobela, Maria Cisowska, Tadeusz Morawiec, Dariusz Skaba, Rafał Wiench

**Affiliations:** 1 Department of Periodontal Diseases and Oral Mucosa Diseases, Faculty of Medical Sciences in Zabrze, Medical University of Silesia, 40-055 Katowice, Polands88998@365.sum.edu.pl (J.F.-R.); rwiench@sum.edu.pl (R.W.); 2 Department of Microbiology and Immunology, Faculty of Medical Sciences in Zabrze, Medical University of Silesia, Jordana 19 Street, 41-808 Zabrze, Poland; amertas@sum.edu.pl (A.M.); ebobela@sum.edu.pl (E.B.); mcisowska@sum.edu.pl (M.C.); 3 Department of Dental Surgery, Faculty of Medical Sciences in Zabrze, Medical University of Silesia, 15 Poniatowskiego Street, 40-055 Katowice, Poland; tmorawiec@sum.edu.pl

**Keywords:** antimicrobial therapy, photosensitizer, curcumin, riboflavin, photodynamic inactivation, oral infections, *Candida albicans*, *Candida glabrata*, *Candida krusei*, *Staphylococcus aureus*, oral microbiome, planktonic cells, yeasts

## Abstract

Oral candidiasis, commonly caused by *Candida* (*C.*) *albicans* and other non-*albicans Candida* species, increases resistance to conventional antifungal therapies. This study aimed to evaluate the in vitro efficacy of antimicrobial photodynamic therapy (aPDT) using a 450 nm diode laser in combination with curcumin and riboflavin against *Candida* spp. and *Staphylococcus* (*S.*) *aureus*. Reference strains of *C. albicans*, *C. glabrata*, *C. krusei*, and *S. aureus* were exposed to aPDT under varying incubation times and laser parameters, then viable microorganism cells (CFU) counts were assessed the microbial reduction, and statistical analyses were performed to evaluate significance. aPDT significantly reduced microbial viability in a time- and dose-dependent manner. Optimal incubation times were 20 min for *Candida* spp. and 10 min for *S. aureus*, with the highest efficacy observed at 400 mW and 120 s irradiation. The photosensitizer or laser alone had no significant antimicrobial effect. Curcumin/riboflavin-mediated aPDT is a promising alternative or adjunctive approach to conventional antimicrobial therapy, particularly for resistant oral infections.

## 1. Introduction

Oral candidiasis is a common opportunistic infection of the mucous membranes, primarily caused by *Candida albicans*, but increasingly by non-albicans *Candida* (NAC) species, especially in immunocompromised individuals [1,2,3]. Colonization and transition to pathogenic states are facilitated by numerous local and systemic risk factors, including denture use, xerostomia, diabetes, corticosteroid therapy, and HIV/AIDS [2,4,5,6]. Rising antifungal resistance—particularly among NAC species—has led to more frequent treatment failures and recurrent infections [7,8,9,10]. Similarly, *Staphylococcus aureus*, a commensal organism and a major pathogen in skin and mucosal infections, presents a growing challenge due to the emergence of multidrug-resistant strains such as MRSA and VRSA [11,12,13,14,15,16,17]. These trends highlight an urgent need for alternative antimicrobial approaches. Antimicrobial photodynamic therapy (aPDT) offers a non-invasive strategy that uses a photosensitizer (PS), light of an appropriate wavelength, and oxygen to generate reactive oxygen species (ROS), which damage microbial cells [18,19,20,21,22,23,24]. aPDT has shown promise against a wide range of pathogens, including fungi and bacteria, without promoting antimicrobial resistance [22,25]. Natural photosensitizers such as curcumin (CUR) and riboflavin (RIB) are particularly attractive due to their low toxicity, selective activity, and compatibility with blue light (420–450 nm), which induces both Type I and Type II photochemical reactions [24,26,27,28]. Curcumin possesses broad-spectrum antimicrobial, anti-inflammatory, and antioxidant properties, and exhibits rapid photobleaching and limited dark toxicity [27,28,29,30,31,32,33,34,35]. Riboflavin similarly demonstrates favorable safety, tissue compatibility, and efficacy across various microbial targets [36,37,38]. Both agents can be activated by 450 nm light, making them suitable for combined use in aPDT. However, despite numerous studies exploring aPDT with curcumin alone, data on CUR-RIB combinations activated by a single laser wavelength, particularly against both *Candida* spp. and *S. aureus*, remain limited [33,34,35,36]. Therefore, the aim of this study was to evaluate the in vitro antimicrobial efficacy of curcumin- and riboflavin-mediated photodynamic therapy using a 450 nm diode laser against reference strains of *C. albicans*, *C. glabrata*, *C. krusei*, and *S. aureus*. We sought to determine the optimal photosensitizer incubation times and laser settings required for effective microbial reduction, including in mixed-species suspensions. The aim of this study was to determine the optimal incubation time required for the absorption of curcumin with riboflavin by cells of selected *Candida* spp. and *S. aureus* strains, and subsequently to evaluate the efficacy of aPDT using different parameters of a 450 nm diode laser and PS against these microorganisms. Unlike previous studies, which typically investigated curcumin-mediated aPDT against either *Candida* spp. or *S. aureus* separately, our study provides a comparative, dose-dependent evaluation of a dual-photosensitizer system (curcumin and riboflavin) combined with a 450 nm diode laser, targeting both fungal and bacterial reference strains—including mixed-species suspensions—to determine optimal incubation times and irradiation parameters in a standardized, reproducible in vitro protocol. This dual-target, dual-PS approach under a single wavelength has not been simultaneously examined in the existing literature.

## 2. Results

### 2.1. Phase 1 Results-Effect of Incubation Time

**Figure 1:**
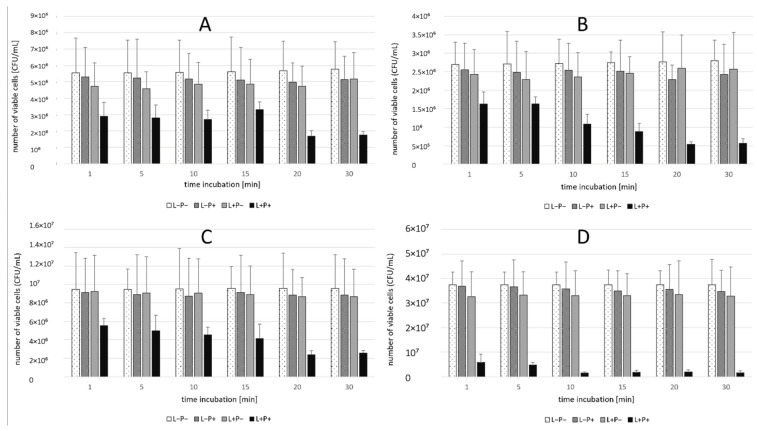
(**A**–**D**) Influence of different incubation times (130 min) on the effect of photodynamic inactivation in a reduction in the number of viable cells [CFU/mL] of different strains in planktonic form. CFU/mL was determined after curcumin/riboflavin-mediated aPDT (L+P+), treatment with light alone (L+P-), or treatment with the photosensitizer alone (L-P+) and compared to negative control treatment (L-P-). Data are mean values and standard deviations from four replicate experiments. (**A**) *C. albicans* ATCC 10231 (**B**) *C. krusei* ATCC 14243 (**C**) *C. glabrata* ATCC 2001 (**D**) *S. aureus* ATCC 25923.

**Figure 2:**
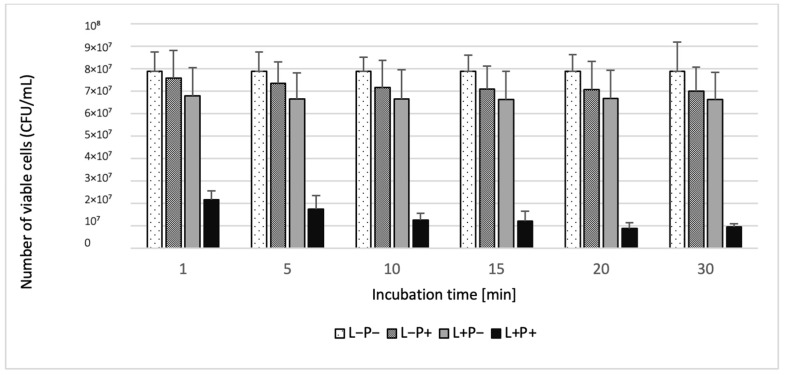
Influence of different incubation times (1–30 min) on the effect of photodynamic inactivation in a reduction in the number of viable cells [CFU/mL] of *C. albicans* ATCC 10231 and *S. aureus* ATCC 25923 in planktonic form. CFU/mL was determined after curcumin/ryboflavin-mediated aPDT (L+P+), treatment with light alone (L+P-), or treatment with the photosensitizer alone (L-P+) and compared to negative control treatment (L-P-). Data are mean values and standard deviations from four replicate experiments.

The antimicrobial effectiveness of aPDT increased with longer photosensitizer incubation times, reaching peak reductions after 20 min for *Candida* spp. and 10 min for *S. aureus*. Among the single-species suspensions, the highest CFU reduction was observed for *S. aureus ATCC 25923* (95.86%) after 10 min, while the lowest significant reduction occurred for *C. glabrata* ATCC 2001 (41.50%) after 1 min. For *Candida* spp., reductions after 20 min ranged from 70.44% (*C. albicans* ATCC 10231) to 80.64% (*C. krusei* ATCC 14243). In the dual-species suspension, the highest reduction (89.05%) was recorded after 20 min (Table 1). The photosensitizer-only (L-P+) and light-only (L+P) control groups showed minimal reductions (<15%) across all species, with no statistically significant differences from the untreated control (L-P-). These findings are summarized in Table 1 and illustrated in Figure 1 and Figure 2.

### 2.2. Phase 2 Results-Effect of Laser Parameters

**Figure 3:**
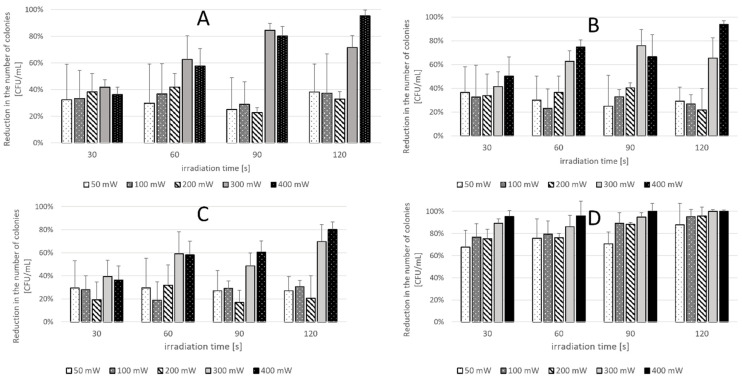
The following study was conducted on the percentage reduction number of viable cells [CFU/mL] of microorganisms in planktonic form, results for the photodynamic group (relative to the control group) using different physical laser settings (output power; irradiation time). Data are mean values and standard deviations from four replicate experiments. (**A**)* C. albicans* ATCC 10231 (**B**) *C. krusei* ATCC 14243 (**C**) *C. glabrata* ATCC 2001 (**D**) *S. aureus* ATCC 25923.

**Figure 4:**
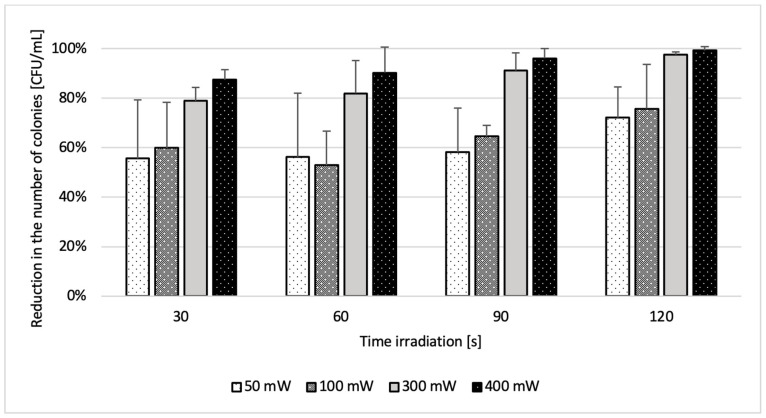
The following study was conducted on the percentage reduction number of viable cells [CFU/mL] of *S. aureus* ATCC 25923 and *C. albicans* ATCC 10231 in planktonic form, results for the photodynamic group (relative to the control group) using different physical laser settings (output power; irradiation time). Data are mean values and standard deviations from four replicate experiments.

**Figure 5:**
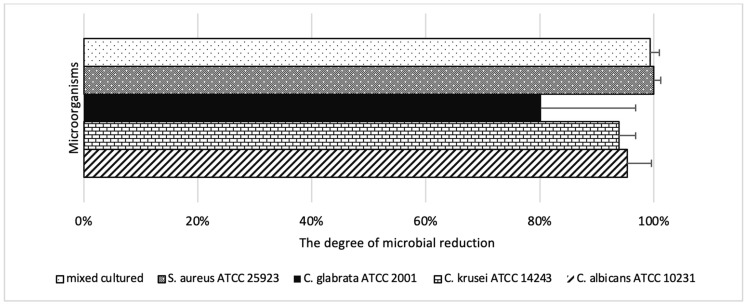
The graph demonstrates the degree of microbial reduction (%) achieved using the optimal laser parameters (output power: 400 mW; irradiation time: 120 s) and the optimal photosensitizer incubation time from Phase I of the experiment for a given species.

In Phase 2, microbial inactivation was laser dose-dependent. The highest reductions occurred at 400 mW and 120 s for all tested strains. *S. aureus* ATCC 25923 showed the most pronounced effect, reaching complete eradication under multiple high-dose conditions. For *C. albicans* ATCC 10231 and *C. krusei* ATCC 14243, reductions of up to 95.32% and 93.90%, respectively, were achieved. *C. glabrata* ATCC 2001 showed the lowest overall response, with a maximum reduction of 80.20%. In the dual-species suspension, the maximum effect was 99.34% under high-fluence settings (Table 2). Minimal reductions (<25%) were observed in the (L+P-) and (L-P+) control groups across all species and settings. Full numerical data are presented in Table 2, Table 3, Table 4, Table 5 and Table 6 and visualized in Figure 3 and Figure 4. In the (L-P+) group, which received the combined curcumin and riboflavin photosensitizer without laser activation, no statistically significant reduction in CFU/mL was observed for any of the tested microorganisms across the 1–30-min incubation range. For *C. albicans* ATCC 10231, reductions ranged from 7.97% to 11.73% (*p* > 0.56); for *C. glabrata* ATCC 2001, from 3.18% to 8.18% (*p* > 0.80); for *C. krusei* ATCC 14243, from 6.96% to 14.58% (*p* > 0.49); and for *S. aureus* ATCC 25923, from 1.13% to 8.49% (*p* > 0.64). Similarly, the dual-species suspension (*C. albicans* ATCC 10231+ *S. aureus* ATCC 25923) showed reductions between 6.23% and 12.22% (*p* = 0.182717). These findings confirm that the photosensitizer formulation alone—without laser activation—exerted minimal antimicrobial activity under the tested conditions, reinforcing the requirement for light activation to achieve significant microbial inactivation in antimicrobial photodynamic therapy. It was demonstrated that the exclusive use of laser (L+P-) resulted in a minimum reduction in microorganisms CFU/mL at: The percentage of *C. albicans* ATCC 10231 ranged from 3.13% to 23.20% (*p* > 0.28), while for *C. krusei* ATCC 14243, the range was from 2.64% to 17.24% (*p* > 0.56). For *C. glabrata* ATCC 2001, the range was from 2.51% to 14.43% (*p* > 0.81). For *S. aureus* ATCC 25923, the lowest score recorded was 3.11%, while the highest score was 14.48% (*p* > 0.45). For the dual suspension (*S. aureus* ATCC 25923 and *C. albicans* ATCC 10231), the lowest score recorded was 3.78%, while the highest score was 18.50% (*p* > 0.19). Figure 5 presents CFU/mL reductions for each microorganism in the (L+P+) group relative to the control (L-P-), under optimal laser parameters.

## 3. Discussion

The optimal incubation time of the photosensitizer (i.e., the time between the addition of the PS to the planktonic solution and the initiation of laser irradiation) is a crucial element of the protocol. It allows sufficient uptake of the PS by the cell wall/membrane and cytoplasm, including organelles, and significantly impacts the effectiveness of photodynamic therapy. Our results confirm this, as the efficacy of aPDT—measured by the reduction in CFU/mL—was evaluated under identical laser settings and PS concentration, with incubation time (1–30 min) being the only variable. Data analysis showed that the optimal incubation time yielding the greatest microbial reduction was 20 min for *C. albicans* ATCC 10231 (70.44%, *p* = 0.000135), *C. krusei* ATCC 14243 (80.64%, *p* = 0.000044), *C. glabrata* ATCC 2001 (75.27%, *p* = 0.000433), and 10 min for *S. aureus* ATCC 25923 (95.86%, *p* = 0.000002). Numerous published studies have evaluated aPDT with curcumin (CUR), including incubation time as a key parameter [18,39,40,41,42,43]. Similar conclusions were drawn by Ma et al., who studied photodynamic therapy with curcumin on the standard *C. albicans* strain ATCC 90028 and two clinical isolates from HIV (CCA1) and oral lichen planus (CCA2) patients. Using a 20-min CUR incubation (irradiation time: 6 min; fluence: 7.92 J/cm^2^), inhibition rates reached 90.87% for ATCC 90028, 66.44% for CCA1, and 86.74% for CCA2 (*p* < 0.05), under optimal laser settings [39]. Patients with immunodeficiencies are more vulnerable to *Candida* infections and face greater challenges in antifungal treatment due to higher drug resistance and impaired host defenses, underscoring the need for alternative therapies [44]. Dovigo et al. also studied the photodynamic effect of CUR against *C. albicans* ATCC 90028, using two incubation times (5 and 20 min). They observed microbial reductions of 68.19% and 87.22%, respectively, with optimal laser parameters. Effective yeast cell reduction was accompanied by minimal deactivation of immune cells, and spectral changes indicated high photobleaching efficiency of curcumin, suggesting minimal phototoxicity [40]. Imaizumi et al. investigated CUR-based aPDT against *S. aureus* ATCC 12600, using a significantly longer incubation time (60 min) than in our study. With 3-min irradiation, they achieved a statistically significant bacterial reduction of 99.99% (*p* = 0.011). Clinically, shorter incubation times are preferred for treating patients [41]. ROS generated by aPDT can attack polyunsaturated fatty acids in bacterial membranes, ultimately causing membrane degradation, increased intracellular ROS, cell rupture, and leakage of intracellular content. ROS also damages DNA, proteins, and enzymes [42]. The addition of 50 µL of CUR/RIB to the (L+P+) and (L-P+) groups allowed for assessment of the photodynamic and photosensitizer-only effects, respectively, while the use of 50 µL of sterile tryptone water in the (L+P) and (L-P-) groups ensured volume consistency across all wells and served as appropriate controls to isolate the effects of light and PS. This setup allowed for direct comparison of the antimicrobial impact of the photosensitizer, the laser alone, and their combined effect under identical experimental conditions.

In contrast to our findings, a study by Al-Asmari et al. showed that incubating yeast cells with CUR for 10, 20, and 30 min before irradiation did not result in significant differences in microbial reduction [45]. Although antimicrobial photodynamic therapy (aPDT) is primarily based on non-thermal photochemical mechanisms, it is recognized that prolonged laser irradiation—particularly beyond 10 s—may induce thermal effects. In our study, thermal buildup was minimized through the use of a 450 nm diode laser fitted with a flat-top handpiece, which ensures uniform energy distribution without hot spots. Additionally, irradiation was conducted in a pulsed fashion (i.e., with each well irradiated sequentially) in a controlled environment, allowing for sufficient passive cooling between exposures. The short exposure time per well (maximum of 120 s) and low total irradiance (≤48 J/cm^2^) further reduced the risk of heat accumulation, and no signs of thermal denaturation or medium evaporation were observed during the experiments.

Another critical factor influencing photodynamic reaction efficacy is the physical parameters of the light source (irradiation time, output power, energy density-fluence). The physical settings of the laser are closely linked to the efficiency of the photodynamic reaction, which is based on the activation of the photosensitizer and the production of ROS, leading to the inactivation of microorganisms. It is imperative to select optimal parameters for the balance between therapeutic efficacy and clinical safety (type of pathogens, location of infection). In our experience, the most favorable applications for all studied species are an irradiation time of 120 s and output power of 400 mW (fluence—48 J/cm^2^). In the Dovigo et al. study, a fluence of 37 J/cm^2^ proved sufficient for the complete elimination of planktonic forms of *C. albicans* [46]. Asmari et al. studied the effect of photodynamic therapy on the inactivation of fungal spores and cells (*C. albicans* ATCC 10231, *A. niger* ATCC 6275, *A. flavus* ATCC 9643, *P. griseofulvum* ATCC 48927, *P. chrysogenum* ATCC 10106, *F. oxysporum* ATCC 62606, and *Z. bailii* ATCC 42476). Significant (*p* < 0.001) fungal reduction was achieved at light doses ranging from 72 to 96 J/cm^2^, corresponding to 6–8 min of exposure to irradiation [45]. In contradistinction to bacteria, fungal spores/cells are surrounded by a thick cell wall (60–80 nm) composed of chitin, α-β glucans, and mannoproteins. The cell wall provides a protective structure against the external environment by limiting diffusion into the cytoplasm. Photosensitization can be subdivided into three distinct stages: initial damage to the cell wall/membrane, cytoplasm (enzymes, RNA, and other subcellular structures), and DNA damage. In comparison to bacteria, fungi are less susceptible to DNA damage due to the presence of a nuclear membrane surrounding their DNA. The process of photoinactivation is contingent upon the uptake or accumulation of photosensitizer within the cellular structures [47,48]. Trigo-Gutierrez et al. conducted a study on photoreactive polymeric micelles to investigate the release of antimicrobial curcumin under blue light at a fluence of 33.84 J/cm^2^ (used an incubation time of 20 min; irradiation time of 12 min). The nanocarriers were successfully utilized to enhance the bioavailability of CUR. In vitro studies conducted by the authors demonstrated that aPDT exhibited the capacity to inactivate the pathogenic microorganisms *C. albicans* SC5314, *P. aeruginosa* ATCC 27853, and methicillin-resistant *Staphylococcus aureus* (MRSA) ATCC 33591. In the context of planktonic *C. albicans*, a statistically significant decline in yeast viability was observed (*p* < 0.001), with a decrease of 1 log_10_ (CFU/mL) in comparison to the untreated control group. A comparable decline was noted for planktonic MRSA, where a decrease of 2 log_10_ (CFU/mL) was recorded (*p* < 0.001), also in relation to the untreated control group. For planktonic *P. aeruginosa*, a decrease of 1 log_10_ (CFU/mL) was observed (*p* ≤ 0.048), once more in comparison to the untreated control group [49].

Sammarro Silva et al. investigated the use of hydrogen peroxide as a strategy to enhance the antimicrobial effectiveness of photodynamic therapy against MRSA. Their results showed a slight reduction in aPDT efficacy at very low concentrations of H_2_O_2_, followed by a synergistic effect of pre-oxidation and aPDT, leading to a significant reduction in MRSA. Complete inactivation of the organisms was observed when the samples were incubated with concentrations > 0.05% H_2_O_2_ [50]. Multidrug resistance is widespread and has increased in recent years, largely due to excessive antibiotic exposure, advancements in laboratory diagnostics, and climate change [51]. Key concerns in managing resistant pathogens include their ability to evade drug mechanisms (e.g., overexpression of target enzymes that remain active in the presence of drugs, upregulation of efflux pumps that reduce intracellular drug levels, mutations reducing drug import), as well as their capacity to escape host immunity. Consequently, exploring alternative therapies is crucial when conventional pharmacological treatments fail [52,53,54].

Curcumin as a photosensitizer in aPDT was studied by Trigo Gutierrez et al. against a dual-species biofilm of *C. albicans* ATCC 90028 and *MRSA* ATCC 33591. Significant reductions in viability were observed for both species: 2.06 log_10_ for *C. albicans* (*p* < 0.001) and 1.39 log_10_ for *MRSA* (*p* = 0.002). aPDT also significantly reduced the viability of all three tested species—*C. albicans* ATCC 90028 (2.41 log_10_), MRSA ATCC 33591 (0.90 log_10_), and *S. mutans* ATCC 700610 (1.05 log_10_)—compared to control and other treatments (*p* = 0.007 for *C. albicans*, *p* = 0.002 for *MRSA*, and *p* < 0.001 for *S. mutans*). The authors used a CUR incubation time of 40 min and irradiation for 20 min, achieving a fluence of 43.2 J/cm^2^. Compared to our results, the degree of microbial reduction was lower, likely due to the treatment limitations imposed by biofilm structure [43]. Biofilm-forming pathogens are significantly more resistant to antimicrobial agents and photosensitizer penetration than planktonic cells. A biofilm is a multicellular structure in which microorganisms are embedded in extracellular polymeric substances (EPSs), which serve structural, nutritional, and protective roles. Effective pathogen inactivation in biofilms is limited due to: reduced penetration of therapeutic agents, drug absorption by EPSs, presence of metabolically inactive subpopulations, horizontal gene transfer of resistance, and drug deactivation within the biofilm environment [55,56]. Standard pharmacotherapy often proves inadequate, necessitating integrated approaches such as combination therapies (e.g., aPDT) [43]. Andrade et al. studied curcumin penetration into biofilms at different incubation times. Using confocal microscopy (Leica TCS SPE, Leica Microsystems GmbH, Wetzlar, Germany) with a 405 nm excitation wavelength and green fluorescence detection (450–600 nm), they observed stronger fluorescence after 20 min of incubation, which correlated with deeper curcumin penetration in monoculture biofilms compared to 5 min. Thus, longer incubation was recommended for effective biofilm elimination [57]. Bakun et al. evaluated the photocytotoxicity and potential utility of aPDT for *S. aureus* NCTC 4163, *E. coli* ATCC 25922, *P. aeruginosa* NCTC 6749, *S. pneumoniae* ATCC 19615, and *C. albicans* ATCC 10231 [58]. Wiench et al. noted that *C. glabrata* employs a unique defense mechanism against TBO (toluidine blue ortho) by aggregating individual cells into large, compact structures, which can prevent PS from reaching cells in the center of the suspension [18]. In the study by Dovigo et al., the complete elimination of *C. albicans* from the tongue mucosa of test mice was observed after a single therapeutic intervention (LED 455 nm, fluence 37.5 J/cm^2^, power density 89.2 mW, and curcumin concentration of 80 µM) [42]. The therapy was effective regardless of the biofilm’s developmental stage. A reduction in yeast cells and hyphae was observed in the biofilm [8]. CUR-mediated aPDT also promoted reduced expression of *C. albicans* genes related to adhesion, biofilm formation, and oxidative stress response [5,18]. However, curcumin’s poor water solubility, instability, and low bioavailability limit its in vivo applications [43]. A further limitation of aPDT is that it can only treat superficial, localized areas accessible to light exposure. The aPDT protocol often requires several minutes of PS incubation before irradiation, which can be difficult to achieve in the oral cavity due to factors such as salivary flow. As a result, attempts are being made to encapsulate curcumin in nanocarriers to enhance its stability or in adhesive formulations to facilitate its application. Despite numerous laboratory studies, the optimal concentrations of photosensitizers and physical parameters of light sources have not yet been established, resulting in the absence of a standardized clinical protocol. The clinical effectiveness of aPDT as a therapeutic method for treating fungal infections of the oral cavity still requires further investigation [18].

The present study may have useful implications for clinical work with patients. The application of the QroxB2 algorithm, involving an incubation period of 15–20 min followed by laser irradiation for 120 s at an output power of 400 mW, has been demonstrated to be a promising treatment modality that can complement conventional therapy and, in certain cases, potentially substitute for it.

Despite demonstrating promising results in vitro, this study has several limitations. First, the experiments were conducted exclusively on planktonic microbial cultures, which do not fully replicate the complex architecture and increased resistance of biofilm-associated infections commonly seen in clinical oral environments. Second, only reference laboratory strains were tested, which may not reflect the full spectrum of resistance and virulence found in clinical isolates. Third, the conditions of the in vitro model—such as controlled temperature, absence of saliva, and static incubation—do not mimic the dynamic and multifactorial environment of the oral cavity, potentially limiting the direct clinical applicability of the findings. Moreover, curcumin’s known instability in aqueous environments and its poor solubility might affect its consistency as a photosensitizer under real-world conditions. Additionally, the need for relatively long photosensitizer incubation times (up to 20 min) and specific irradiation protocols may pose practical challenges in clinical settings. Finally, while the study showed significant microbial reductions, complete eradication was not achieved in all cases, and the potential cytotoxic effects of the treatment on host tissues were not assessed, necessitating further preclinical and clinical investigations.

The findings of this study suggest that curcumin- and riboflavin-mediated antimicrobial photodynamic therapy (aPDT) using a 450 nm diode laser could become a valuable adjunct or alternative to conventional antifungal and antibacterial treatments, particularly in cases of drug-resistant oral infections. The demonstrated efficacy against both *Candida* spp. and *S. aureus*, combined with the use of natural, low-toxicity photosensitizers and non-invasive light therapy, offers a promising therapeutic strategy for clinicians managing recurrent or refractory oral mucosal infections. The optimal parameters identified—20-min photosensitizer incubation for *Candida* spp., 10 min for *S. aureus,* and laser irradiation at 400 mW for 120 s—provide a structured protocol that could be adapted for clinical settings. Nevertheless, clinical application will require addressing practical challenges, such as ensuring adequate photosensitizer retention in the dynamic oral environment and evaluating the therapy’s effectiveness against biofilm-associated infections. These promising in vitro results warrant further in vivo studies and clinical trials to refine protocols and confirm safety, efficacy, and feasibility before widespread implementation in dental and medical practice.

## 4. Materials and Methods

The study was conducted at the Microbiological Laboratory of Silesia LabMed Research and Implementation Center in the Department of Microbiology and Immunology, Faculty of Medical Sciences in Zabrze, Medical University of Silesia in Katowice.

### 4.1. Reference Microbial Strains

The study was performed using three reference strains of *Candida* spp. obtained from the ATCC (American Type Culture Collection, Manassas, VA, USA): *C. albicans* ATCC 10231, *C. glabrata* ATCC 2001, *C. krusei* ATCC 14243, as well as a reference strain of *S. aureus* ATCC 25923. The *Candida* strains were cultured on Sabouraud agar (bioMérieux SA, Marcy l’Étoile, France), and the *S. aureus* strain was cultured on Columbia agar supplemented with 5% sheep blood (bioMérieux SA, Marcy l’Étoile, France). Microbial cultures were incubated at 37 °C and passaged every 48–72 h according to ATCC guidelines. Microorganisms used in the study were taken from 24-h cultures to prepare working suspensions with a density of 3.0 × 10^8^ CFU/mL in sterile 0.9% NaCl solution. Suspension density was measured using a Densi-La-Meter II laboratory densitometer (Erba Polska Sp. z o.o., Kraków, Poland).

### 4.2. Photosensitizer and Laser

The photosensitizer QroxB2 (CMS Dental, Roslev, Denmark) was utilized in the present study. The product under discussion is of a commercial nature, intended for medical use, and contains both curcumin and riboflavin (CUR/RIB) in its formulation at concentrations of 1%. It is a yellowish dry powder packaged in a single-use syringe. Before use, the powder is dissolved in sterile demineralized water. QroxB2 is sealed in an airtight aluminum pouch. The light source was a 450 nm diode laser (PIOON S1 Blue, PIOON, Wuhan, China) operating in continuous wave (CW) mode, with an applicator surface of approximately 0.5 cm^2^ (8 mm diameter), fitted with a flat-top tip ensuring uniform energy distribution across the laser beam (i.e., no “hot spot” as in the classical Gaussian power distribution). Laser power settings were 50/100/200/300/400 mW with irradiation times of 30/60/90/120 s, yielding energy densities (fluence) of 1.5–48 J/cm^2^. During irradiation, temperature rise was minimized by conducting the procedure within a laminar flow cabinet at room temperature, using a diode laser with a flat-top beam profile to avoid hot spots. Each well was irradiated individually with brief pauses between samples to allow passive heat dissipation, preventing cumulative thermal effects.

### 4.3. First Phase of the Study

Evaluation of the optimal incubation time of curcumin/riboflavin in experimental groups for reducing viable cells of individual microorganisms under constant laser parameters.

A total of 384 tests were conducted (96 per tested microorganism), divided into four experimental groups:

1. (L+P+) aPDT group—suspension exposed to both photosensitizer and laser (*n* = 4);
2. (L-P+) Photosensitizer group—suspension treated with the PS only, without laser exposure (*n* = 4);
3. (L+P-) Light-only group—suspension exposed to laser only, without PS (*n* = 4);
4. (L-P-) Control group—suspension not exposed to laser or PS (*n* = 4).

In each group, the effect of different incubation times of CUR/RIB (1/5/10/15/20/30 min) was assessed using fixed laser parameters (output power: 400 mW; irradiation time: 60 s) on the reduction in viable microorganism cells CFU/mL in the suspensions. In each experimental group, *n* = 4 indicates that four independent replicates were performed for each condition, ensuring statistical reliability and reproducibility of the results.

To experiment, 200 µL of the working microbial suspension was added to selected 24 wells of black, sterile, 96-well microtiter plates with lids (Thermo Fisher Scientific, Waltham, MA, USA), leaving one empty well between samples to prevent cross-diffusion of light. Then, 50 µL of CUR/RIB was added to the wells in groups (L+P+) and (L-P+), while 50 µL of tryptone water was added to the wells in groups (L+P-) and (L-P-). The plates were shaken for 1 min at 350 rpm at 35 °C using a thermal shaker (PST-60 HL-4, Biosan, Riga, Latvia). In the dark, at room temperature, and within a class II laminar flow cabinet (BIO ACTIVA VE 120, AQUARIA SRL, Lacchiarella, Italy), the lid was removed, and each well was irradiated (sequentially) after the respective incubation time (1/5/10/15/20/30 min) according to the protocol for groups (L+P+) and (L+P-). During irradiation, the laser tip was mounted on a stand positioned 1 mm above the well surface. The other wells were covered with a black matte screen with a hole matching the applicator tip’s diameter to prevent light from spreading to adjacent wells. Immediately after irradiation, 10 µL of the well’s suspension was transferred into a tube containing 4 mL of tryptone water. After mixing, 10 µL of the diluted suspension was plated (in duplicate) onto Sabouraud agar and incubated for 48 h at 35 °C (for *Candida* spp.) or onto Columbia agar with 5% sheep blood and incubated for 24 h at 37 °C (for *Staphylococcus aureus*). The same procedure was followed for the remaining groups according to the study protocol. After incubation, microbial colonies were counted using an automated colony counter (ProtoCOL 3, Synbiosis, Cambridge, UK), and the number of viable cells in experimental suspensions was calculated to assess the reduction in microbial cell count because of the photosensitizer and laser.

### 4.4. Second Phase of the Study

Experimental Groups and Photodynamic Inactivation of *Candida* spp. and *Staphylococcus aureus* in vitro.

A total of 1280 tests were performed (320 per tested microorganism), divided into four experimental groups:

1. (L+P+) aPDT group—suspension exposed to both the photosensitizer and laser (*n* = 4);
2. (L-P+) Photosensitizer group—suspension treated with the PS only, without laser exposure (*n* = 4);
3. (L+P-) Light-only group—suspension exposed to laser only, without PS (*n* = 4);
4. (L-P-) Control group—suspension not exposed to either the laser or PS (*n* = 4).

In each group, the effect of different laser settings (output power and irradiation time) on the reduction in viable cells CFU/mL in the microbial suspensions was assessed, using the optimal incubation times for each microorganism as determined in Phase I of the study. The laser power settings applied were 50/100/200/300/400 mW, with exposure times of 30/60/90/120 s. This resulted in an energy density (fluence) of 1.5–48 J/cm^2^.

To experiment, 200 µL of the working suspension of a given microorganism was added to selected 24 wells of black, sterile 96-well microtiter plates with lids (Thermo Fisher Scientific, Waltham, MA, USA), with one empty well left between samples to prevent cross-light diffusion. Then, 50 µL of CUR/RIB was added to the wells of groups (L+P+) and (L-P+), while 50 µL of tryptone water was added to wells in groups (L+P-) and (L-P-). The plates were shaken for 1 min at 350 rpm at 35 °C, using a thermal shaker (PST-60 HL-4, Biosan, Riga, Latvia).

In the dark, at room temperature, and inside a Class II laminar flow cabinet (BIO ACTIVA VE 120, AQUARIA SRL, Lacchiarella, Italy), the plate lids were removed, and the wells were irradiated one at a time at the appropriate incubation time—20 min for *Candida* spp. and 10 min for *S. aureus*—following the described protocol for groups (L+P+) and (L+P-). During irradiation, the laser (with a flat-tip applicator) was mounted on a stand positioned 1 mm above the cell suspension ssurface. The remaining wells were covered with a black matte screen with an opening corresponding to the diameter of the laser applicator tip, preventing the spread of light to adjacent wells. Immediately after irradiation, 10 µL of the well’s suspension was transferred into a tube containing 4 mL of tryptone water. After mixing, 10 µL of the diluted microbial suspension was plated in duplicate onto Sabouraud agar and incubated for 48 h at 35 °C (*Candida* spp.) or onto Columbia agar with 5% sheep blood and incubated for 24 h at 37 °C (*Staphylococcus aureus*). The same procedure was followed for the remaining experimental groups according to the study protocol. After incubation, microbial colonies were counted using an automatic colony counter (ProtoCOL 3, Synbiosis, Cambridge, UK), and the number of viable microbial cells in the experimental suspensions was calculated to assess the reduction in cell counts resulting from the combined effect of the photosensitizer and laser.

### 4.5. Statistical Analysis

Statistical analysis was performed using Statistica software, version 13.3 (StatSoft, Warsaw, Poland). The initial step in the statistical analysis was to ascertain the normality of the data distribution using the Shapiro–Wilk test. Subsequently, the Levene test was employed to ascertain the homogeneity of variances. The following text is intended to provide a comprehensive overview of the subject matter. The NIR test was utilized to conduct comparative analyses between the designated study groups. Statistically significant differences in the evaluated parameters were considered to have occurred when *p* < 0.05.

## 5. Conclusions

This study confirms that curcumin- and riboflavin-mediated antimicrobial photodynamic therapy (aPDT) using a 450 nm diode laser effectively reduces *Candida* spp. and *S. aureus* in vitro. Optimal microbial reduction was achieved with specific incubation times (15–20 min for *Candida*, 10 min for *S. aureus*) and high laser settings (400 mW, 120 s). The combined use of photosensitizer and light was essential, as neither alone produced significant effects. These findings highlight the potential of aPDT as a promising adjunct to conventional antimicrobial treatments, warranting further research for clinical application and protocol standardization.

## Figures and Tables

**Table 1:** Maximum and minimum CFU reductions (%) by incubation time for each strain (Phase 1).

Microorganism	Max Reduction (%)	Time (min)	Min Reduction (%)	Time (min)
*S. aureus* ATCC 25923	95.9	10	843	1
*C. krusei* ATCC 14243	80.6	20	39.8	1
*C. glabrata* ATCC 2001	75.3	20	41.5	1
*C. albicans* ATCC 10231	70.4	20	41.3	15
*S. aureus* + *C. albicans* mix	89.1	20	72.7	1

**Table 2:** Maximum and minimum CFU reductions (%) by laser settings for each strain (Phase 2).

Microorganism	Max Reduction (%)	Power (mW)	Time (s)	Min Reduction (%)	Power (mW)	Time (s)
*S. aureus* ATCC 25923	100.0	300–400	90–120	67.6	50	30
*C. albicans* ATCC 10231	95.3	400	120	25.1	50	90
*C. krusei* ATCC 14243	93.9	400	120	23.3	100	60
*C. glabrata* ATCC 2001	80.2	400	120	19.0	100	60
*S. aureus* + *C. albicans* mix	99.3	400	120	52.9	100	60

**Table 3:** The following study was conducted on the percentage reduction number of viable cells [CFU/mL] of *C. albicans* ATCC 10231 in planktonic form, results for the photodynamic group (relative to the control group) using different physical laser settings (output power; irradiation time).

*C. albicans* ATCC 10231	50 mW	100 mW	200 mW	300 mW	400 mW
30 s	32.4%	33.4%	38.2%	41.8%	36.3%
60 s	29.7%	36.8%	41.9%	62.6%	57.7%
90 s	25.1%	28.9%	22.8%	84.5%	80.2%
120 s	38.2%	37.4%	32.9%	71.6%	95.3%

**Table 4:** The following study was conducted on the percentage reduction number of viable cells [CFU/mL] of *C. krusei* ATCC 14243 in planktonic form, results for the photodynamic group (relative to the control group) using different physical laser settings (output power; irradiation time).

*C. krusei* ATCC 14243	50 mW	100 mW	200 mW	300 mW	400 mW
30 s	36.5%	32.7%	33.8%	41.5%	50.3%
60 s	30.1%	23.3%	36.5%	62.7%	74.7%
90 s	25.1%	32.9%	40.3%	75.9%	66.6%
120 s	29.3%	26.8%	21.9%	65.6%	93.9%

**Table 5:** The following study was conducted on the percentage reduction number of viable cells [CFU/mL] of *C. glabrata* ATCC 2001 in planktonic form, results for the photodynamic group (relative to the control group) using different physical laser settings (output power; irradiation time).

*C. glabrata* ATCC 2001	50 mW	100 mW	200 mW	300 mW	400 mW
30 s	29.4%	28.0%	19.3%	39.4%	36.5%
60 s	29.6%	19.0%	31.9%	59.2%	58.1%
90 s	27.0%	29.2%	16.9%	48.5%	60.7%
120 s	27.0%	30.7%	20.7%	69.6%	80.2%

**Table 6:** The following study was conducted on the percentage reduction number of viable cells [CFU/mL] of *S. aureus* ATCC 25923 and *C. albicans* ATCC 10231 in planktonic form, results for the photodynamic group (relative to the control group) using different physical laser settings (output power; irradiation time).

Mixed Cultured	50 mW	100 mW	200 mW	300 mW	400 mW
30 s	55.7%	59.9%	66.7%	78.9%	87.5%
60 s	56.3%	52.9%	57.8%	81.9%	90.3%
90 s	58.2%	64.6%	76.0%	91.2%	96.2%
120 s	72.1%	75.8%	81.8%	97.6%	99.3%

## Data Availability

Data are available on request from the corresponding author.
